# Supplementary figures and images for: Media and social media attention to retracted articles according to Altmetric
Source: PLoS One. 2021 May 12;16(5):e0248625. doi: 10.1371/journal.pone.0248625 (PMC8115781; doi:10.1371/journal.pone.0248625)

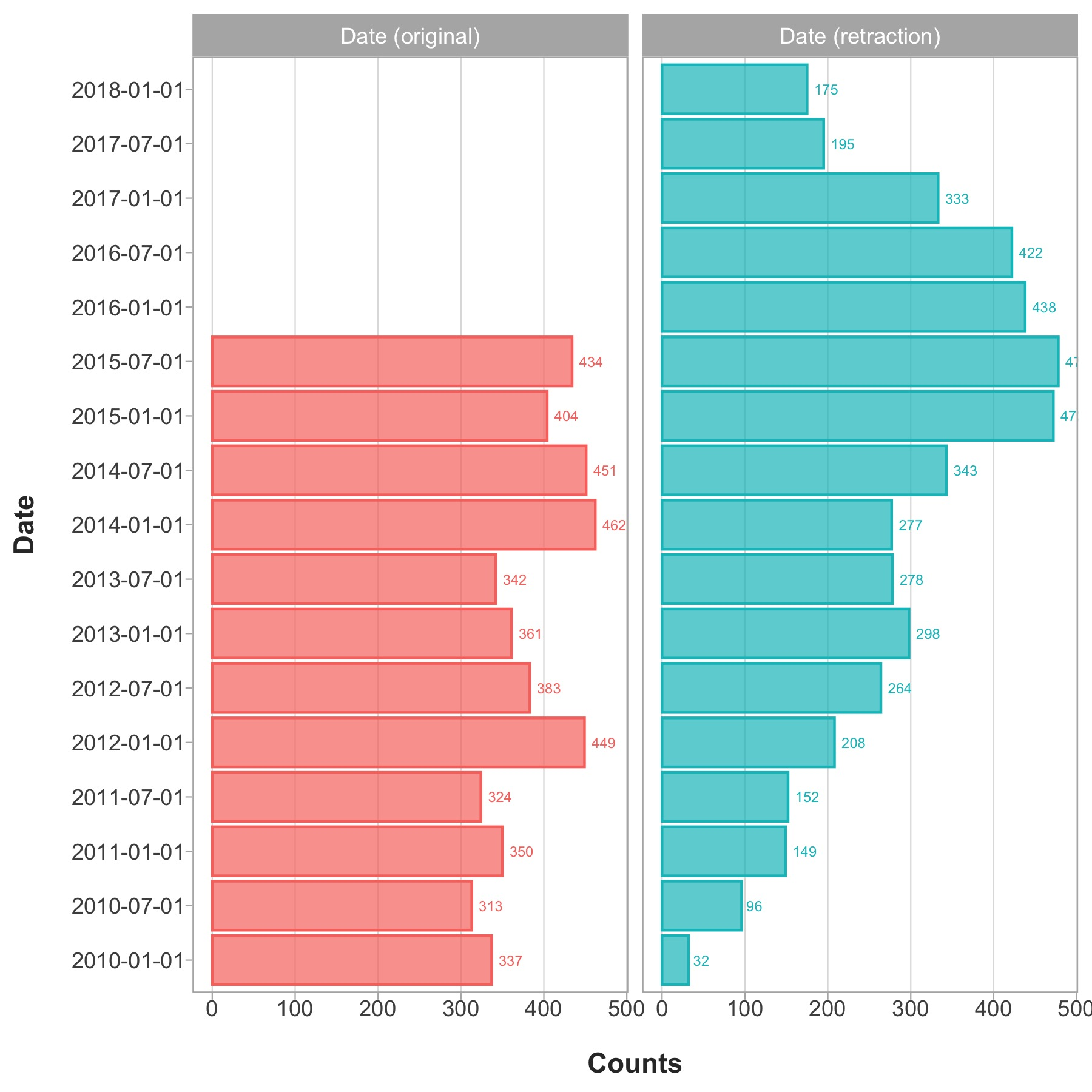

Supplement: S1 Fig — The distribution of retracted literature is roughly uniform across 6-month periods, whereas the distribution of their retractions follows a bell-curve with a left skew. This skew reflects that more publications are retracted very early rather than very late. (TIF) [file pone.0248625.s001.tif]

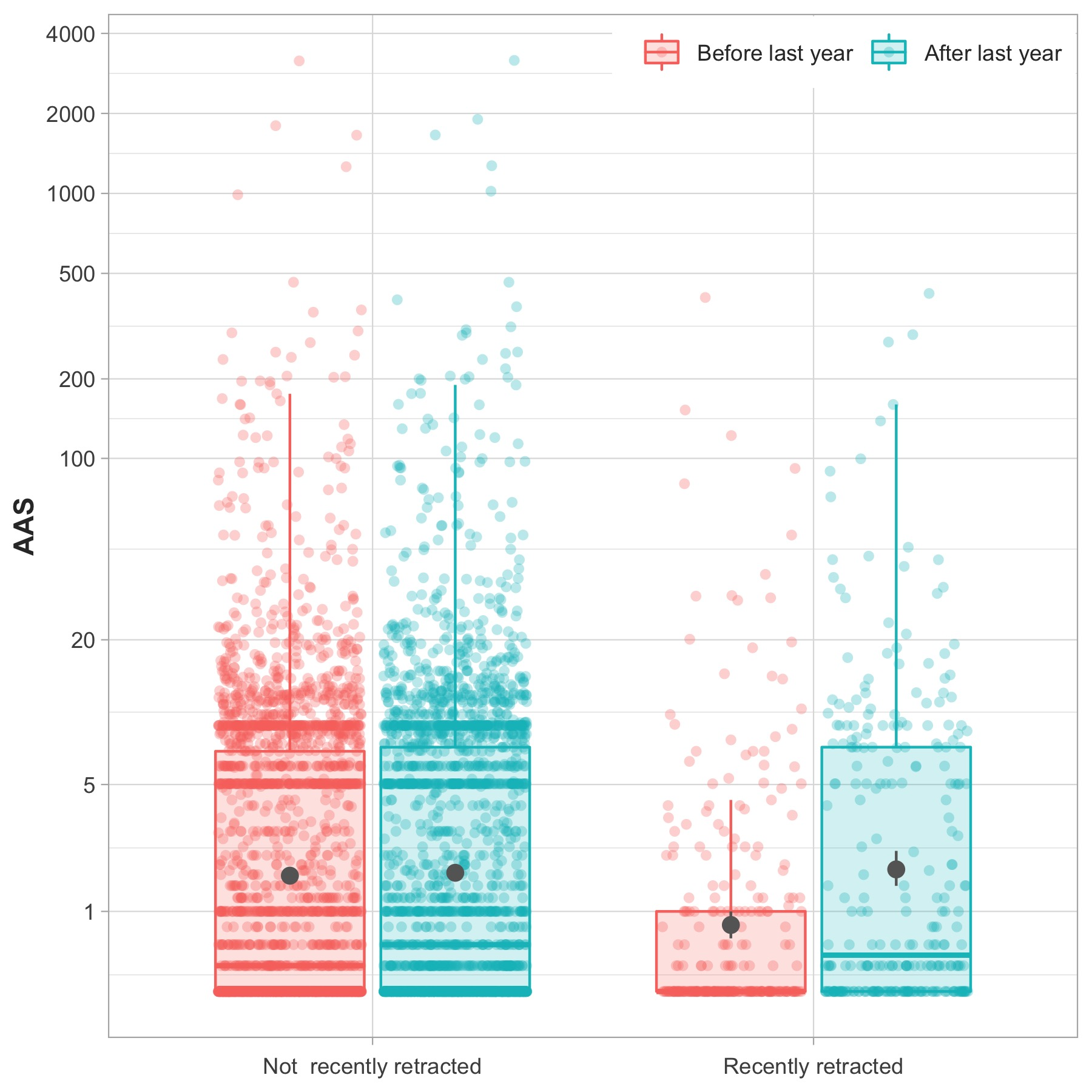

Supplement: S2 Fig — There is no meaningful difference between the distribution of 2,733 articles retracted more than a year ago between AAS received before versus after the last year. On the contrary, for the 275 articles retracted within the last year, there is marked increase in total AAS when considering the last year. This implies that a substantial proportion of the total attention is received after retraction. The distribution of total AAS in articles that were recently retracted versus not, is not meaningfully different. The vertical axis is transformed to reflect log(AAS + 1). The grey dots are the mean and its bootstrapped 95% CI. (TIF) [file pone.0248625.s002.tif]

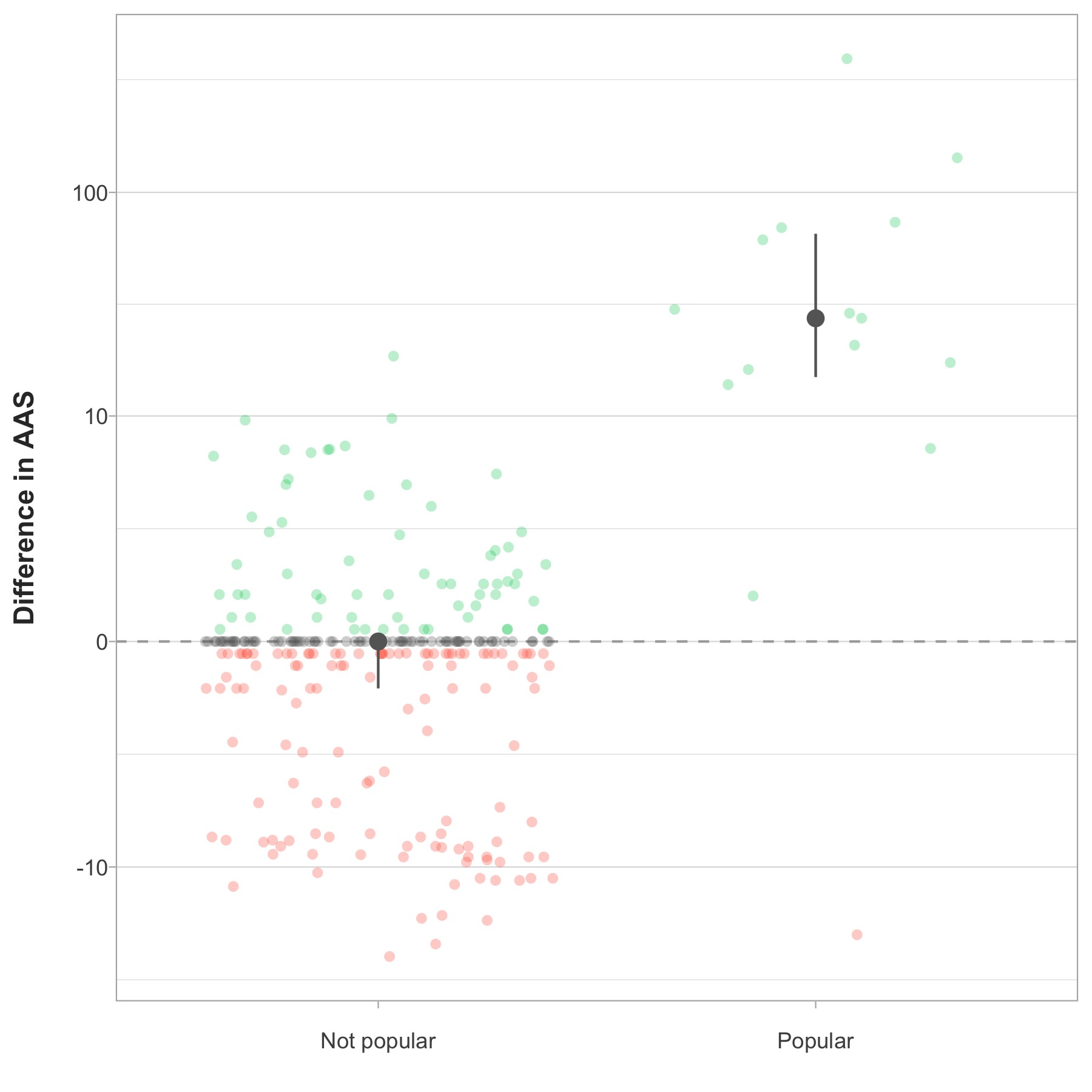

Supplement: S3 Fig — All points in green represent an original article that received more media and social media attention than its retraction notice and all points in red represent the opposite; points in grey represent no difference between the two. The large point and solid line in grey represent the median and its interquartile range. The difference is rather balanced for 260 articles that are not popular. In 15 popular articles, the difference is skewed to the right such that most popular articles did not have an equally popular retraction. (TIF) [file pone.0248625.s003.tif]
