# Supplementary material for: Media and social media attention to retracted articles according to Altmetric
Source: PLoS One. 2021 May 12;16(5):e0248625. doi: 10.1371/journal.pone.0248625 (PMC8115781; doi:10.1371/journal.pone.0248625)
Supplement: S1 Table — (HTML) [file pone.0248625.s004.html]

Data Frame Summary


### Data Frame Summary

#### RetractionWatch

**Dimensions**: 22226 x 29
  
**Duplicates**: 0
  

| **No** | **Variable** | **Stats / Values** | **Freqs (% of Valid)** | **Graph** | **Valid** | **Missing** |
| --- | --- | --- | --- | --- | --- | --- |
| 1 | id [numeric] | Mean (sd) : 11754 (6876) min < med < max: 1 < 11744 < 23998 IQR (CV) : 11764 (0.6) | 22226 distinct values |  | 22226 (100.0%) | 0 (0.0%) |
| 2 | id\_group [numeric] | Mean (sd) : 11100 (6408) min < med < max: 1 < 11102 < 22200 IQR (CV) : 11100 (0.6) | 22200 distinct values |  | 22226 (100.0%) | 0 (0.0%) |
| 3 | is\_eligible [logical] | 1. FALSE 2. TRUE | |  |  |  |  | | --- | --- | --- | --- | | 17609 | ( | 79.2% | ) | | 4611 | ( | 20.8% | ) | |  | 22220 (100.0%) | 6 (0.0%) |
| 4 | is\_research [logical] | 1. FALSE 2. TRUE | |  |  |  |  | | --- | --- | --- | --- | | 9576 | ( | 43.1% | ) | | 12650 | ( | 56.9% | ) | |  | 22226 (100.0%) | 0 (0.0%) |
| 5 | is\_popular [logical] | 1. FALSE 2. TRUE | |  |  |  |  | | --- | --- | --- | --- | | 6738 | ( | 97.0% | ) | | 208 | ( | 3.0% | ) | |  | 6946 (31.3%) | 15280 (68.7%) |
| 6 | title [character] | 1. CIP2A mediates prostate c 2. 3-Benzhydryl-4-piperidone 3. A cough induced pelvic fr 4. A hybrid CFD framework fo 5. A method of multi-criteri 6. A new deep representation 7. Analysis of security oper 8. Assessing Efficacy of Lip 9. Association of cytokine g 10. Blockade of Receptor for [ 22168 others ] | |  |  |  |  | | --- | --- | --- | --- | | 3 | ( | 0.0% | ) | | 2 | ( | 0.0% | ) | | 2 | ( | 0.0% | ) | | 2 | ( | 0.0% | ) | | 2 | ( | 0.0% | ) | | 2 | ( | 0.0% | ) | | 2 | ( | 0.0% | ) | | 2 | ( | 0.0% | ) | | 2 | ( | 0.0% | ) | | 2 | ( | 0.0% | ) | | 22205 | ( | 99.9% | ) | |  | 22226 (100.0%) | 0 (0.0%) |
| 7 | subject [character] | 1. (PHY) Mathematics 2. (B/T) Computer Science; ( 3. (B/T) Computer Science 4. (PHY) Chemistry 5. (B/T) Business - Manageme 6. (B/T) Business - Economic 7. (PHY) Engineering - Elect 8. (PHY) Materials Science 9. (SOC) Education 10. (BLS) Biochemistry; (BLS) [ 6646 others ] | |  |  |  |  | | --- | --- | --- | --- | | 285 | ( | 1.3% | ) | | 283 | ( | 1.3% | ) | | 266 | ( | 1.2% | ) | | 244 | ( | 1.1% | ) | | 214 | ( | 1.0% | ) | | 208 | ( | 0.9% | ) | | 192 | ( | 0.9% | ) | | 182 | ( | 0.8% | ) | | 175 | ( | 0.8% | ) | | 173 | ( | 0.8% | ) | | 20004 | ( | 90.0% | ) | |  | 22226 (100.0%) | 0 (0.0%) |
| 8 | institute [character] | 1. Department of Anesthesiol 2. Department of Materials S 3. Plasma Physics Research C 4. Romania 5. Electrical and Computer E 6. College of Chemistry and 7. College of Engineering, J 8. College of Chemical Engin 9. Department of Anaesthesio 10. Department of Anesthesiol [ 20441 others ] | |  |  |  |  | | --- | --- | --- | --- | | 19 | ( | 0.1% | ) | | 15 | ( | 0.1% | ) | | 15 | ( | 0.1% | ) | | 15 | ( | 0.1% | ) | | 14 | ( | 0.1% | ) | | 13 | ( | 0.1% | ) | | 13 | ( | 0.1% | ) | | 10 | ( | 0.0% | ) | | 10 | ( | 0.0% | ) | | 10 | ( | 0.0% | ) | | 21684 | ( | 99.4% | ) | |  | 21818 (98.2%) | 408 (1.8%) |
| 9 | journal [character] | 1. 2011 International Confer 2. 2010 International Confer 3. Journal of Fundamental an 4. 2010 IEEE International C 5. 2011 2nd Intl Conf on Art 6. 2010 2nd International Co 7. The Journal of Biological 8. 2010 The 2nd Conference o 9. PLoS One 10. 2009 International Confer [ 4971 others ] | |  |  |  |  | | --- | --- | --- | --- | | 1281 | ( | 5.8% | ) | | 440 | ( | 2.0% | ) | | 434 | ( | 2.0% | ) | | 428 | ( | 1.9% | ) | | 403 | ( | 1.8% | ) | | 359 | ( | 1.6% | ) | | 335 | ( | 1.5% | ) | | 290 | ( | 1.3% | ) | | 281 | ( | 1.3% | ) | | 274 | ( | 1.2% | ) | | 17701 | ( | 79.6% | ) | |  | 22226 (100.0%) | 0 (0.0%) |
| 10 | publisher [character] | 1. Institute of Electrical a 2. Elsevier 3. Springer 4. Wiley 5. Taylor and Francis 6. University of El Qued 7. SAGE Publications 8. American Society for Bioc 9. PLoS 10. Wolters Kluwer [ 880 others ] | |  |  |  |  | | --- | --- | --- | --- | | 7498 | ( | 33.7% | ) | | 2962 | ( | 13.3% | ) | | 1689 | ( | 7.6% | ) | | 1121 | ( | 5.0% | ) | | 616 | ( | 2.8% | ) | | 434 | ( | 2.0% | ) | | 394 | ( | 1.8% | ) | | 343 | ( | 1.5% | ) | | 301 | ( | 1.4% | ) | | 287 | ( | 1.3% | ) | | 6581 | ( | 29.6% | ) | |  | 22226 (100.0%) | 0 (0.0%) |
| 11 | country [character] | 1. China 2. United States 3. India 4. Japan 5. Iran 6. South Korea 7. Germany 8. Unknown 9. United Kingdom 10. Italy [ 989 others ] | |  |  |  |  | | --- | --- | --- | --- | | 9587 | ( | 43.2% | ) | | 2352 | ( | 10.6% | ) | | 1011 | ( | 4.6% | ) | | 800 | ( | 3.6% | ) | | 641 | ( | 2.9% | ) | | 484 | ( | 2.2% | ) | | 405 | ( | 1.8% | ) | | 366 | ( | 1.6% | ) | | 362 | ( | 1.6% | ) | | 293 | ( | 1.3% | ) | | 5913 | ( | 26.6% | ) | |  | 22214 (99.9%) | 12 (0.1%) |
| 12 | author [character] | 1. Chandra Gopala Rao 2. Adrian Maxim 3. Richard Lawrence Etienne 4. Soon-Gi Shin 5. Ali Nazari; Shadi Riahi 6. Thomas M Rosica 7. Yoshitaka Fujii; Hiroyosh 8. Spyros P Panagopoulos 9. Yoshitaka Fujii; Yuhji Sa 10. Robert James Cardullo [ 20726 others ] | |  |  |  |  | | --- | --- | --- | --- | | 52 | ( | 0.2% | ) | | 26 | ( | 0.1% | ) | | 26 | ( | 0.1% | ) | | 26 | ( | 0.1% | ) | | 24 | ( | 0.1% | ) | | 22 | ( | 0.1% | ) | | 21 | ( | 0.1% | ) | | 20 | ( | 0.1% | ) | | 20 | ( | 0.1% | ) | | 19 | ( | 0.1% | ) | | 21958 | ( | 98.8% | ) | |  | 22214 (99.9%) | 12 (0.1%) |
| 13 | urls [character] | 1. http://retractionwatch.co 2. http://retractionwatch.co 3. http://retractionwatch.co 4. http://retractionwatch.co 5. http://retractionwatch.co 6. http://retractionwatch.co 7. http://retractionwatch.co 8. http://retractionwatch.co 9. http://retractionwatch.co 10. http://retractionwatch.co [ 2971 others ] | |  |  |  |  | | --- | --- | --- | --- | | 6646 | ( | 53.8% | ) | | 434 | ( | 3.5% | ) | | 106 | ( | 0.9% | ) | | 106 | ( | 0.9% | ) | | 92 | ( | 0.7% | ) | | 49 | ( | 0.4% | ) | | 38 | ( | 0.3% | ) | | 32 | ( | 0.3% | ) | | 29 | ( | 0.2% | ) | | 28 | ( | 0.2% | ) | | 4795 | ( | 38.8% | ) | |  | 12355 (55.6%) | 9871 (44.4%) |
| 14 | article\_type [character] | 1. Research Article 2. Conference Abstract/Paper 3. Clinical Study 4. Review Article 5. Case Report 6. Meta-Analysis 7. Book Chapter/Reference Wo 8. Commentary/Editorial 9. Article in Press; Researc 10. Clinical Study; Research [ 56 others ] | |  |  |  |  | | --- | --- | --- | --- | | 10973 | ( | 49.4% | ) | | 7954 | ( | 35.8% | ) | | 1126 | ( | 5.1% | ) | | 832 | ( | 3.7% | ) | | 317 | ( | 1.4% | ) | | 185 | ( | 0.8% | ) | | 129 | ( | 0.6% | ) | | 104 | ( | 0.5% | ) | | 69 | ( | 0.3% | ) | | 65 | ( | 0.3% | ) | | 472 | ( | 2.1% | ) | |  | 22226 (100.0%) | 0 (0.0%) |
| 15 | original\_date [POSIXct, POSIXt] | min : 1923-01-01 med : 2011-05-06 max : 2020-08-01 range : 97y 7m 0d | 4949 distinct values |  | 22173 (99.8%) | 53 (0.2%) |
| 16 | original\_doi [character] | 1. 10.1007/s11042-017-5385-3 2. 10.1001/archpediatrics.20 3. 10.1007/s00113-005-0932-1 4. 10.1007/s00125-015-3687-4 5. 10.1007/s10865-012-9399-z 6. 10.1007/s11042-017-5403-5 7. 10.1007/s11042-017-5557-1 8. 10.1007/s11242-012-9973-4 9. 10.1007/s11427-007-0101-6 10. 10.1007/s13277-014-2995-5 [ 16796 others ] | |  |  |  |  | | --- | --- | --- | --- | | 3 | ( | 0.0% | ) | | 2 | ( | 0.0% | ) | | 2 | ( | 0.0% | ) | | 2 | ( | 0.0% | ) | | 2 | ( | 0.0% | ) | | 2 | ( | 0.0% | ) | | 2 | ( | 0.0% | ) | | 2 | ( | 0.0% | ) | | 2 | ( | 0.0% | ) | | 2 | ( | 0.0% | ) | | 16834 | ( | 99.9% | ) | |  | 16855 (75.8%) | 5371 (24.2%) |
| 17 | original\_pubmed [numeric] | Mean (sd) : 21159540 (6848757) min < med < max: 993 < 22198782 < 32764974 IQR (CV) : 9036195 (0.3) | 8812 distinct values |  | 8856 (39.8%) | 13370 (60.2%) |
| 18 | retract\_date [POSIXct, POSIXt] | min : 1927-04-01 med : 2012-06-22 max : 2020-08-14 range : 93y 4m 13d | 3898 distinct values |  | 22225 (100.0%) | 1 (0.0%) |
| 19 | retract\_doi [character] | 1. 10.1007/s13277-017-5487-6 2. 10.1177/1077546314541924 3. 10.2174/18744443016080101 4. 10.1107/s1600536809049964 5. 10.1107/s1600536809054300 6. 10.1016/s0925-4773(03)000 7. 10.1107/s1600536809049976 8. 10.1107/s1600536811037603 9. 10.1177/1056789519875280 10. 10.3109/08923973.2012.710 [ 19442 others ] | |  |  |  |  | | --- | --- | --- | --- | | 107 | ( | 0.5% | ) | | 60 | ( | 0.3% | ) | | 43 | ( | 0.2% | ) | | 40 | ( | 0.2% | ) | | 39 | ( | 0.2% | ) | | 38 | ( | 0.2% | ) | | 29 | ( | 0.1% | ) | | 28 | ( | 0.1% | ) | | 22 | ( | 0.1% | ) | | 20 | ( | 0.1% | ) | | 19927 | ( | 97.9% | ) | |  | 20353 (91.6%) | 1873 (8.4%) |
| 20 | retract\_pubmed [numeric] | Mean (sd) : 24501259 (6411294) min < med < max: 195582 < 25679970 < 32779578 IQR (CV) : 7893553 (0.3) | 8038 distinct values |  | 8542 (38.4%) | 13684 (61.6%) |
| 21 | notice [character] | 1. Retraction | |  |  |  |  | | --- | --- | --- | --- | | 22226 | ( | 100.0% | ) | |  | 22226 (100.0%) | 0 (0.0%) |
| 22 | reason [character] | 1. Notice - Limited or No In 2. Breach of Policy by Autho 3. Duplication of Article 4. Notice - Limited or No In 5. Plagiarism of Article 6. Investigation by Journal/ 7. Investigation by Journal/ 8. Notice - Limited or No In 9. Duplication of Article; E 10. Breach of Policy by Autho [ 3688 others ] | |  |  |  |  | | --- | --- | --- | --- | | 4174 | ( | 18.8% | ) | | 1318 | ( | 5.9% | ) | | 976 | ( | 4.4% | ) | | 639 | ( | 2.9% | ) | | 607 | ( | 2.7% | ) | | 558 | ( | 2.5% | ) | | 452 | ( | 2.0% | ) | | 391 | ( | 1.8% | ) | | 368 | ( | 1.7% | ) | | 316 | ( | 1.4% | ) | | 12427 | ( | 55.9% | ) | |  | 22226 (100.0%) | 0 (0.0%) |
| 23 | paywalled [character] | 1. No 2. Unknown 3. Yes | |  |  |  |  | | --- | --- | --- | --- | | 20484 | ( | 92.8% | ) | | 217 | ( | 1.0% | ) | | 1375 | ( | 6.2% | ) | |  | 22076 (99.3%) | 150 (0.7%) |
| 24 | notes [character] | 1. exact date of retraction 2. Journal published by SAGE 3. See also IEEE conference 4. See also RW post about ot 5. Journal formerly Acta Cry 6. see also: https://www.iee 7. 42774 8. exact date of retraction 9. Chen-Yuan 'Peter' Chen wa 10. Chen-Yuan 'Peter' Chen wa [ 1995 others ] | |  |  |  |  | | --- | --- | --- | --- | | 182 | ( | 5.8% | ) | | 102 | ( | 3.2% | ) | | 58 | ( | 1.8% | ) | | 45 | ( | 1.4% | ) | | 41 | ( | 1.3% | ) | | 29 | ( | 0.9% | ) | | 23 | ( | 0.7% | ) | | 23 | ( | 0.7% | ) | | 21 | ( | 0.7% | ) | | 18 | ( | 0.6% | ) | | 2614 | ( | 82.8% | ) | |  | 3156 (14.2%) | 19070 (85.8%) |
| 25 | discipline [character] | 1. Biological sciences; Heal 2. Physical sciences 3. Biological sciences 4. Business and Technology 5. Health sciences 6. Business and Technology; 7. Business and Technology; 8. Social sciences 9. Business and Technology; 10. Environmental sciences; P [ 63 others ] | |  |  |  |  | | --- | --- | --- | --- | | 3533 | ( | 15.9% | ) | | 3135 | ( | 14.1% | ) | | 2915 | ( | 13.1% | ) | | 2597 | ( | 11.7% | ) | | 2372 | ( | 10.7% | ) | | 2124 | ( | 9.6% | ) | | 1041 | ( | 4.7% | ) | | 595 | ( | 2.7% | ) | | 543 | ( | 2.4% | ) | | 351 | ( | 1.6% | ) | | 3020 | ( | 13.6% | ) | |  | 22226 (100.0%) | 0 (0.0%) |
| 26 | original\_aas [numeric] | Mean (sd) : 5.7 (57.8) min < med < max: 0 < 0 < 3166 IQR (CV) : 1.2 (10.2) | 482 distinct values |  | 6946 (31.3%) | 15280 (68.7%) |
| 27 | original\_cite [numeric] | Mean (sd) : 5.7 (16.1) min < med < max: 0 < 1 < 280 IQR (CV) : 5 (2.8) | 124 distinct values |  | 6879 (31.0%) | 15347 (69.0%) |
| 28 | retract\_aas [numeric] | Mean (sd) : 7.1 (43.7) min < med < max: 0 < 0 < 482 IQR (CV) : 0 (6.2) | 356 distinct values |  | 9586 (43.1%) | 12640 (56.9%) |
| 29 | retract\_cite [numeric] | Mean (sd) : 0.5 (2.4) min < med < max: 0 < 0 < 75 IQR (CV) : 0 (5.2) | 38 distinct values |  | 9505 (42.8%) | 12721 (57.2%) |
