# Supplementary material for: Media and social media attention to retracted articles according to Altmetric
Source: PLoS One. 2021 May 12;16(5):e0248625. doi: 10.1371/journal.pone.0248625 (PMC8115781; doi:10.1371/journal.pone.0248625)
Supplement: S2 Table — (HTML) [file pone.0248625.s005.html]

Data Frame Summary


### Data Frame Summary

#### Eligible research articles

**Dimensions**: 4611 x 29
  
**Duplicates**: 0
  

| **No** | **Variable** | **Stats / Values** | **Freqs (% of Valid)** | **Graph** | **Valid** | **Missing** |
| --- | --- | --- | --- | --- | --- | --- |
| 1 | id [numeric] | Mean (sd) : 8237 (6442) min < med < max: 1 < 6031 < 23799 IQR (CV) : 11953 (0.8) | 4611 distinct values |  | 4611 (100.0%) | 0 (0.0%) |
| 2 | id\_group [numeric] | Mean (sd) : 10728 (6059) min < med < max: 4 < 10385 < 22200 IQR (CV) : 9546 (0.6) | 4603 distinct values |  | 4611 (100.0%) | 0 (0.0%) |
| 3 | is\_eligible [logical] | 1. TRUE | |  |  |  |  | | --- | --- | --- | --- | | 4611 | ( | 100.0% | ) | |  | 4611 (100.0%) | 0 (0.0%) |
| 4 | is\_research [logical] | 1. TRUE | |  |  |  |  | | --- | --- | --- | --- | | 4611 | ( | 100.0% | ) | |  | 4611 (100.0%) | 0 (0.0%) |
| 5 | is\_popular [logical] | 1. FALSE 2. TRUE | |  |  |  |  | | --- | --- | --- | --- | | 3661 | ( | 95.0% | ) | | 192 | ( | 5.0% | ) | |  | 3853 (83.6%) | 758 (16.4%) |
| 6 | title [character] | 1. CIP2A mediates prostate c 2. A hybrid CFD framework fo 3. Blockade of Receptor for 4. Can Branding Improve Scho 5. Development of synthetic 6. Growth of polynomials wit 7. Mass analysis by Ar-GCIB- 8. Measles-mumps-rubella Vac 9. Preference for immediate 10. Pressure Transient Analys [ 4589 others ] | |  |  |  |  | | --- | --- | --- | --- | | 3 | ( | 0.1% | ) | | 2 | ( | 0.0% | ) | | 2 | ( | 0.0% | ) | | 2 | ( | 0.0% | ) | | 2 | ( | 0.0% | ) | | 2 | ( | 0.0% | ) | | 2 | ( | 0.0% | ) | | 2 | ( | 0.0% | ) | | 2 | ( | 0.0% | ) | | 2 | ( | 0.0% | ) | | 4590 | ( | 99.5% | ) | |  | 4611 (100.0%) | 0 (0.0%) |
| 7 | subject [character] | 1. (PHY) Mathematics 2. (PHY) Chemistry 3. (B/T) Computer Science; ( 4. (PHY) Materials Science 5. (BLS) Biochemistry; (BLS) 6. (PHY) Chemistry; (PHY) Ma 7. (B/T) Computer Science 8. (PHY) Engineering - Elect 9. (PHY) Physics 10. (PHY) Chemistry; (PHY) Ph [ 2287 others ] | |  |  |  |  | | --- | --- | --- | --- | | 90 | ( | 2.0% | ) | | 83 | ( | 1.8% | ) | | 64 | ( | 1.4% | ) | | 58 | ( | 1.3% | ) | | 44 | ( | 1.0% | ) | | 40 | ( | 0.9% | ) | | 34 | ( | 0.7% | ) | | 32 | ( | 0.7% | ) | | 32 | ( | 0.7% | ) | | 30 | ( | 0.7% | ) | | 4104 | ( | 89.0% | ) | |  | 4611 (100.0%) | 0 (0.0%) |
| 8 | institute [character] | 1. Department of Advanced Ma 2. Department of Chemistry, 3. Department of Mathematics 4. Gorgan University of Agri 5. Institute of Maritime Inf 6. Office of the University 7. Romania 8. a9.com, 101 Lytton Avenue 9. Department of Molecular B 10. Department of Structural, [ 4412 others ] | |  |  |  |  | | --- | --- | --- | --- | | 8 | ( | 0.2% | ) | | 7 | ( | 0.2% | ) | | 6 | ( | 0.1% | ) | | 6 | ( | 0.1% | ) | | 5 | ( | 0.1% | ) | | 5 | ( | 0.1% | ) | | 5 | ( | 0.1% | ) | | 4 | ( | 0.1% | ) | | 4 | ( | 0.1% | ) | | 4 | ( | 0.1% | ) | | 4515 | ( | 98.8% | ) | |  | 4569 (99.1%) | 42 (0.9%) |
| 9 | journal [character] | 1. Tumor Biology (Tumour Bio 2. The Journal of Biological 3. PLoS One 4. The Open Automation and C 5. Journal of Vibration and 6. Molecular Biology Reports 7. Diagnostic Pathology 8. PNAS: Proceedings of the 9. Archives of Biological Sc 10. Immunopharmacology and Im [ 2229 others ] | |  |  |  |  | | --- | --- | --- | --- | | 146 | ( | 3.2% | ) | | 88 | ( | 1.9% | ) | | 73 | ( | 1.6% | ) | | 48 | ( | 1.0% | ) | | 47 | ( | 1.0% | ) | | 32 | ( | 0.7% | ) | | 31 | ( | 0.7% | ) | | 24 | ( | 0.5% | ) | | 20 | ( | 0.4% | ) | | 20 | ( | 0.4% | ) | | 4082 | ( | 88.5% | ) | |  | 4611 (100.0%) | 0 (0.0%) |
| 10 | publisher [character] | 1. Elsevier 2. Springer 3. Wiley 4. Taylor and Francis 5. SAGE Publications 6. BioMed Central (BMC) 7. Hindawi 8. American Society for Bioc 9. Wolters Kluwer 10. PLoS [ 382 others ] | |  |  |  |  | | --- | --- | --- | --- | | 940 | ( | 20.4% | ) | | 722 | ( | 15.7% | ) | | 333 | ( | 7.2% | ) | | 249 | ( | 5.4% | ) | | 154 | ( | 3.3% | ) | | 134 | ( | 2.9% | ) | | 112 | ( | 2.4% | ) | | 90 | ( | 2.0% | ) | | 83 | ( | 1.8% | ) | | 82 | ( | 1.8% | ) | | 1712 | ( | 37.1% | ) | |  | 4611 (100.0%) | 0 (0.0%) |
| 11 | country [character] | 1. China 2. United States 3. India 4. Iran 5. South Korea 6. Japan 7. Germany 8. Italy 9. Taiwan 10. China; United States [ 468 others ] | |  |  |  |  | | --- | --- | --- | --- | | 1071 | ( | 23.2% | ) | | 571 | ( | 12.4% | ) | | 341 | ( | 7.4% | ) | | 268 | ( | 5.8% | ) | | 181 | ( | 3.9% | ) | | 134 | ( | 2.9% | ) | | 96 | ( | 2.1% | ) | | 92 | ( | 2.0% | ) | | 85 | ( | 1.8% | ) | | 83 | ( | 1.8% | ) | | 1688 | ( | 36.6% | ) | |  | 4610 (100.0%) | 1 (0.0%) |
| 12 | author [character] | 1. Soon-Gi Shin 2. Richard Lawrence Etienne 3. Bahram Mokhtari; Kobra Po 4. Cheng-Wu Chen 5. Mariusz Ksiazek 6. Feng Wang; Yong Yang 7. Mohammad Alibakhshi Kenar 8. Nataly Shulga; John G Pas 9. Bojidarka Ivanova; Michae 10. Janus S Liang [ 4380 others ] | |  |  |  |  | | --- | --- | --- | --- | | 19 | ( | 0.4% | ) | | 8 | ( | 0.2% | ) | | 7 | ( | 0.2% | ) | | 6 | ( | 0.1% | ) | | 6 | ( | 0.1% | ) | | 5 | ( | 0.1% | ) | | 5 | ( | 0.1% | ) | | 5 | ( | 0.1% | ) | | 4 | ( | 0.1% | ) | | 4 | ( | 0.1% | ) | | 4541 | ( | 98.5% | ) | |  | 4610 (100.0%) | 1 (0.0%) |
| 13 | urls [character] | 1. http://retractionwatch.co 2. http://retractionwatch.co 3. http://retractionwatch.co 4. http://retractionwatch.co 5. http://retractionwatch.co 6. http://retractionwatch.co 7. http://retractionwatch.co 8. http://retractionwatch.co 9. http://retractionwatch.co 10. http://retractionwatch.co [ 1520 others ] | |  |  |  |  | | --- | --- | --- | --- | | 104 | ( | 4.6% | ) | | 32 | ( | 1.4% | ) | | 27 | ( | 1.2% | ) | | 27 | ( | 1.2% | ) | | 23 | ( | 1.0% | ) | | 19 | ( | 0.8% | ) | | 11 | ( | 0.5% | ) | | 11 | ( | 0.5% | ) | | 11 | ( | 0.5% | ) | | 11 | ( | 0.5% | ) | | 1983 | ( | 87.8% | ) | |  | 2259 (49.0%) | 2352 (51.0%) |
| 14 | article\_type [character] | 1. Research Article 2. Clinical Study 3. Meta-Analysis 4. Clinical Study; Research 5. Article in Press; Researc 6. Letter 7. Letter; Research Article 8. Case Report; Research Art 9. Case Report; Clinical Stu 10. Article in Press; Clinica [ 12 others ] | |  |  |  |  | | --- | --- | --- | --- | | 3966 | ( | 86.0% | ) | | 406 | ( | 8.8% | ) | | 115 | ( | 2.5% | ) | | 34 | ( | 0.7% | ) | | 32 | ( | 0.7% | ) | | 20 | ( | 0.4% | ) | | 7 | ( | 0.2% | ) | | 6 | ( | 0.1% | ) | | 5 | ( | 0.1% | ) | | 3 | ( | 0.1% | ) | | 17 | ( | 0.4% | ) | |  | 4611 (100.0%) | 0 (0.0%) |
| 15 | original\_date [POSIXct, POSIXt] | min : 2010-01-01 med : 2013-03-07 max : 2015-12-31 range : 5y 11m 30d | 1614 distinct values |  | 4611 (100.0%) | 0 (0.0%) |
| 16 | original\_doi [character] | 1. 10.1001/archpediatrics.20 2. 10.1007/s10865-012-9399-z 3. 10.1007/s11242-012-9973-4 4. 10.1007/s13277-014-2995-5 5. 10.1016/j.cej.2012.06.143 6. 10.1080/15376494.2011.563 7. 10.1097/jom.0b013e3181d8d 8. 10.1186/2047-9158-3-16 9. 10.2106/jbjs.j.00781 10. 10.2337/db12-0317 [ 4304 others ] | |  |  |  |  | | --- | --- | --- | --- | | 2 | ( | 0.0% | ) | | 2 | ( | 0.0% | ) | | 2 | ( | 0.0% | ) | | 2 | ( | 0.0% | ) | | 2 | ( | 0.0% | ) | | 2 | ( | 0.0% | ) | | 2 | ( | 0.0% | ) | | 2 | ( | 0.0% | ) | | 2 | ( | 0.0% | ) | | 2 | ( | 0.0% | ) | | 4304 | ( | 99.5% | ) | |  | 4324 (93.8%) | 287 (6.2%) |
| 17 | original\_pubmed [numeric] | Mean (sd) : 23364282 (2821914) min < med < max: 1994808 < 23624419 < 31482768 IQR (CV) : 3287846 (0.1) | 2680 distinct values |  | 2687 (58.3%) | 1924 (41.7%) |
| 18 | retract\_date [POSIXct, POSIXt] | min : 2010-01-01 med : 2015-03-26 max : 2018-05-29 range : 8y 4m 28d | 1572 distinct values |  | 4611 (100.0%) | 0 (0.0%) |
| 19 | retract\_doi [character] | 1. 10.1007/s13277-017-5487-6 2. 10.1177/1077546314541924 3. 10.2174/18744443016080101 4. 10.3109/08923973.2012.710 5. 10.5812/ircmj.43586 6. 10.1177/0267659115588021 7. 10.1177/1470320315623881 8. 10.1007/2fs11059-014-0289 9. 10.1017/s1759078716001392 10. 10.1515/rjdnmd-2017-0010 [ 3926 others ] | |  |  |  |  | | --- | --- | --- | --- | | 105 | ( | 2.5% | ) | | 45 | ( | 1.1% | ) | | 43 | ( | 1.0% | ) | | 20 | ( | 0.5% | ) | | 10 | ( | 0.2% | ) | | 9 | ( | 0.2% | ) | | 8 | ( | 0.2% | ) | | 7 | ( | 0.2% | ) | | 5 | ( | 0.1% | ) | | 5 | ( | 0.1% | ) | | 3974 | ( | 93.9% | ) | |  | 4231 (91.8%) | 380 (8.2%) |
| 20 | retract\_pubmed [numeric] | Mean (sd) : 25320675 (3122261) min < med < max: 2064438 < 25763425 < 32667144 IQR (CV) : 3709419 (0.1) | 2497 distinct values |  | 2581 (56.0%) | 2030 (44.0%) |
| 21 | notice [character] | 1. Retraction | |  |  |  |  | | --- | --- | --- | --- | | 4611 | ( | 100.0% | ) | |  | 4611 (100.0%) | 0 (0.0%) |
| 22 | reason [character] | 1. Duplication of Article 2. Fake Peer Review; Investi 3. Plagiarism of Article 4. Notice - Limited or No In 5. Duplication of Article; E 6. Plagiarism of Text 7. Notice - Limited or No In 8. Euphemisms for Plagiarism 9. Euphemisms for Plagiarism 10. Duplication of Article; E [ 1623 others ] | |  |  |  |  | | --- | --- | --- | --- | | 316 | ( | 6.9% | ) | | 262 | ( | 5.7% | ) | | 192 | ( | 4.2% | ) | | 126 | ( | 2.7% | ) | | 95 | ( | 2.1% | ) | | 71 | ( | 1.5% | ) | | 68 | ( | 1.5% | ) | | 66 | ( | 1.4% | ) | | 58 | ( | 1.3% | ) | | 54 | ( | 1.2% | ) | | 3303 | ( | 71.6% | ) | |  | 4611 (100.0%) | 0 (0.0%) |
| 23 | paywalled [character] | 1. No 2. Unknown 3. Yes | |  |  |  |  | | --- | --- | --- | --- | | 4212 | ( | 92.4% | ) | | 61 | ( | 1.3% | ) | | 286 | ( | 6.3% | ) | |  | 4559 (98.9%) | 52 (1.1%) |
| 24 | notes [character] | 1. exact date of retraction 2. Journal published by SAGE 3. Chen-Yuan 'Peter' Chen wa 4. exact date of retraction 5. Chen-Yuan 'Peter' Chen wa 6. 42787 7. 42774 8. 42773 9. Chen-Yuan 'Peter' Chen wa 10. 1. Article withdrawn by a [ 510 others ] | |  |  |  |  | | --- | --- | --- | --- | | 100 | ( | 11.0% | ) | | 100 | ( | 11.0% | ) | | 20 | ( | 2.2% | ) | | 20 | ( | 2.2% | ) | | 12 | ( | 1.3% | ) | | 10 | ( | 1.1% | ) | | 9 | ( | 1.0% | ) | | 8 | ( | 0.9% | ) | | 8 | ( | 0.9% | ) | | 7 | ( | 0.8% | ) | | 612 | ( | 67.5% | ) | |  | 906 (19.6%) | 3705 (80.4%) |
| 25 | discipline [character] | 1. Biological sciences; Heal 2. Physical sciences 3. Biological sciences 4. Health sciences 5. Business and Technology 6. Business and Technology; 7. Biological sciences; Envi 8. Biological sciences; Phys 9. Social sciences 10. Environmental sciences; P [ 43 others ] | |  |  |  |  | | --- | --- | --- | --- | | 1244 | ( | 27.0% | ) | | 817 | ( | 17.7% | ) | | 799 | ( | 17.3% | ) | | 580 | ( | 12.6% | ) | | 221 | ( | 4.8% | ) | | 134 | ( | 2.9% | ) | | 98 | ( | 2.1% | ) | | 96 | ( | 2.1% | ) | | 94 | ( | 2.0% | ) | | 67 | ( | 1.5% | ) | | 461 | ( | 10.0% | ) | |  | 4611 (100.0%) | 0 (0.0%) |
| 26 | original\_aas [numeric] | Mean (sd) : 9.5 (76.9) min < med < max: 0 < 0.2 < 3166 IQR (CV) : 7 (8.1) | 446 distinct values |  | 3853 (83.6%) | 758 (16.4%) |
| 27 | original\_cite [numeric] | Mean (sd) : 9.3 (19.8) min < med < max: 0 < 3 < 280 IQR (CV) : 9 (2.1) | 118 distinct values |  | 3794 (82.3%) | 817 (17.7%) |
| 28 | retract\_aas [numeric] | Mean (sd) : 17 (67.8) min < med < max: 0 < 0.2 < 482 IQR (CV) : 8.9 (4) | 339 distinct values |  | 3764 (81.6%) | 847 (18.4%) |
| 29 | retract\_cite [numeric] | Mean (sd) : 0.9 (3.6) min < med < max: 0 < 0 < 75 IQR (CV) : 0 (4) | 38 distinct values |  | 3697 (80.2%) | 914 (19.8%) |
