# Supplementary material for: Media and social media attention to retracted articles according to Altmetric
Source: PLoS One. 2021 May 12;16(5):e0248625. doi: 10.1371/journal.pone.0248625 (PMC8115781; doi:10.1371/journal.pone.0248625)
Supplement: S3 Table — Count = number retracted for specific reason; Percent = proportion retracted for specific reason. Note that the proportions add up to >100% because articles could be retracted for more than one reason. (DOCX) [file pone.0248625.s006.docx]

# S3 Table. Top 20 reasons for retraction for 4,603 unique retracted research articles

| **Reason of retraction (Top 20 out of 86 distinct reasons)** | **Count** | **Percent** |
| --- | --- | --- |
| Duplication of Article | 667 | 14.5% |
| Fake Peer Review | 594 | 12.9% |
| Plagiarism of Article | 412 | 9.0% |
| Error in Data | 369 | 8.0% |
| Unreliable Results | 363 | 7.9% |
| Plagiarism of Text | 339 | 7.4% |
| Notice - Limited or No Information | 329 | 7.1% |
| Misconduct by Author | 314 | 6.8% |
| Duplication of Image | 296 | 6.4% |
| Euphemisms for Plagiarism | 271 | 5.9% |
| Concerns/Issues About Data | 257 | 5.6% |
| Error in Methods | 250 | 5.4% |
| Falsification/Fabrication of Data | 250 | 5.4% |
| Withdrawal | 246 | 5.3% |
| Error in Results and/or Conclusions | 237 | 5.1% |
| Manipulation of Images | 209 | 4.5% |
| Misconduct - Official Investigation/Finding | 194 | 4.2% |
| Error in Analyses | 193 | 4.2% |
| Concerns/Issues About Authorship | 188 | 4.1% |
| Euphemisms for Duplication | 185 | 4.0% |
