# Supplementary material for: Media and social media attention to retracted articles according to Altmetric
Source: PLoS One. 2021 May 12;16(5):e0248625. doi: 10.1371/journal.pone.0248625 (PMC8115781; doi:10.1371/journal.pone.0248625)
Supplement: S4 Table — The “Total” columns compare the total AAS received by the original articles against the total AAS received by their retraction notice. The “Without last” columns compare the AAS received by the original articles without the last year versus the total AAS received by their retraction notice. The values in parentheses are the IQR for the median and the standard deviation for the mean. The p-value is from a Binomial test of articles with greater original vs. retraction attention. Not all articles for “Total” qualified for “Without last”. (DOCX) [file pone.0248625.s007.docx]

# S4 Table. Pairwise comparison of original vs. retraction notice with or without counting the last year of Altmetric attention to the original.

|  | **Overall** | | **Original ≥ 20 AAS** | | **Original > 0 AAS** | |
| --- | --- | --- | --- | --- | --- | --- |
|  | **Total** | **Without last** | **Total** | **Without last** | **Total** | **Without last** |
|  | N = 279 | N = 279 | N = 20 | N = 12 | N = 179 | N = 115 |
| **Median difference (IQR)** | 0 (-0.3-1.5) | 0 (-1-0.1) | 31 (21-82) | 28 (14-71) | 0.8 (0-5) | 0.5 (-1.6-1.9) |
| **Median ratio (IQR)** | 1.4 (0.4-24.7) | 0.3 (0-2.2) | 4.1 (2.8-16.2) | 2.8 (2.0-9.5) | 2.5 (1.0-100.6) | 1.8 (0.5-27.0) |
|  |  |  |  |  |  |  |
| **Original > Retraction** | 121 (43%) | 70 (25%) | 20 (100%) | 11 (92%) | 121 (68%) | 70 (61%) |
| **Retraction > Original** | 82 (29%) | 126 (45%) | 0 (0%) | 1 (8%) | 42 (23%) | 40 (35%) |
| **Equal** | 76 (27%) | 83 (30%) | 0 (0%) | 0 (0%) | 16 (9%) | 5 (4%) |
| **P-value** | 0.007 | 8 x 10^-5^ | 2 x 10^-6^ | 0.006 | 5 x 10^-10^ | 0.005 |
