# Supplementary material for: Media and social media attention to retracted articles according to Altmetric
Source: PLoS One. 2021 May 12;16(5):e0248625. doi: 10.1371/journal.pone.0248625 (PMC8115781; doi:10.1371/journal.pone.0248625)
Supplement: S5 Table — The “Total” columns compare the total AAS received by the original article against the total AAS received by their retraction notice. The “Pre-retraction” columns compare the AAS received by the original article before retraction versus the attention received by its retraction notice plus any post-retraction attention directed to the original article. The values in parentheses are the IQR for the median and the standard deviation for the mean. The p-value is from a Binomial test of articles with greater original vs. retraction attention. Not all articles for “Total” qualified for “Pre-retraction”. (DOCX) [file pone.0248625.s008.docx]

# S5 Table. Pairwise comparison of original article vs. retraction notice when considering total post-retraction AAS.

|  | **Overall** | | **Original ≥ 20 AAS** | | **Original > 0 AAS** | |
| --- | --- | --- | --- | --- | --- | --- |
|  | **Total** | **Pre-retraction** | **Total** | **Pre-retraction** | **Total** | **Pre-retraction** |
|  | N = 279 | N = 279 | N = 20 | N = 15 | N = 179 | N = 124 |
| **Median difference (IQR)** | 0 (-0.3-1.5) | 0.3 (-6.4-0) | 31 (21-82) | 22 (3-55) | 0.8 (0-5) | 0.3 (-8-2) |
| **Median ratio (IQR)** | 1.4 (0.4-24.7) | 0.2 (0-2.2) | 4.1 (2.8-16.2) | 2.4 (1.1-7.9) | 2.5 (1.0-100.6) | 1.4 (0.3-51) |
|  |  |  |  |  |  |  |
| **Original > Retraction** | 121 (43%) | 65 (23%) | 20 (100%) | 12 (80%) | 121 (68%) | 65 (52%) |
| **Retraction > Original** | 82 (29%) | 151 (54%) | 0 (0%) | 3 (20%) | 42 (23%) | 56 (45%) |
| **Equal** | 76 (27%) | 63 (23%) | 0 (0%) | 0 | 16 (9%) | 3 (3%) |
| **P-value** | 0.007 | 5 x 10^-9^ | 2 x 10^-6^ | 0.035 | 5 x 10^-10^ | 0.47 |
