# Supplementary material for: Media and social media attention to retracted articles according to Altmetric
Source: PLoS One. 2021 May 12;16(5):e0248625. doi: 10.1371/journal.pone.0248625 (PMC8115781; doi:10.1371/journal.pone.0248625)
Supplement: Attachment — Submitted filename: response-to-reviewers.docx [file pone.0248625.s009.docx]

# Journal Requirements

When submitting your revision, we need you to address these additional requirements.

1. Please ensure that your manuscript meets PLOS ONE's style requirements, including those for file naming. The PLOS ONE style templates can be found [here](https://journals.plos.org/plosone/s/file?id=wjVg/PLOSOne_formatting_sample_main_body.pdf) and [here](https://journals.plos.org/plosone/s/file?id=ba62/PLOSOne_formatting_sample_title_authors_affiliations.pdf).

*Ensured.*

2. In your Methods section, please include additional information about your dataset and ensure that you have included a statement specifying whether the collection method complied with the terms and conditions for the websites from which you have collected data.

*We have included a Data and Code availability statement and we have also noted that our data were obtained using a data use agreement in compliance with terms and conditions: “This is a retrospective cohort study of the retracted literature found in the Retraction Watch Database shared with us under a data use agreement on August 21, 2020 in compliance with their terms and conditions.”*

3. Please indicate if the authors of the tweets included in Fig. 6 were contacted and agreed for their tweets to be reproduced in this paper. Alternatively, please confirm with your IRB, data protection committee or other relevant research ethics committee that consent from these authors is not required.

*Thank you for alerting us to this concern. Given that all relevant scientific value is already contained within the text, we have now decided to remove Fig.6 and all verbatim mentions of tweets.*

# Reviewer 1

Thank you for the opportunity to review this manuscript. Overall, this approach to the topic of retractions is very timely and innovative.

However, as we detail below, the authors appear to have relied on a dataset that was clearly marked “beta” at the time they extracted the data (May 2018) and was not launched until October 2018. At the very least, that fact should be clearly referenced throughout the manuscript (and the supplementary information, publication of which violates the data use agreement scholars have agreed to in exchange for the data). But more to the point, that seems to throw into doubt the accuracy and reproducibility of the findings. We would suggest that the authors contact us -- the creators of the Retraction Watch Database -- for a complete download of the dataset, at which point they can repeat their analyses with confidence.

*We thank their reviewers for their extremely important observation and we apologize for inadvertently having used a beta version of the data. Indeed, we had identified many inconsistencies in that data ourselves and had gone through a lot of manual work to resolve them. We have now worked with the reviewers, who kindly shared with us the updated version of the Retraction Watch on 21st August, 2020. We hereby present the same analyses using this updated version of the database and clarify that this data is only accessible by request from Retraction Watch in our Data Availability statement:*

*“Data extracted from Altmetric, CrossRef and all matched PubMed articles have been deposited on OSF (Open Science Framework) and may be accessed at https://www.doi.org/10.17605/OSF.IO/7T32U under a CC-By Attribution 4.0 License. The Retraction Watch Database is available from Retraction Watch and requests for this data should be sent to: team@retractionwatch.com.”*

Specific:

**Introduction**

Page 4: “Retraction refers to the formal withdrawal of a peer-reviewed publication, mostly due to …” the referenced citation does not have a stipulation for “peer-reviewed, and to be accurate the “mostly due to” should probably read “most often due to”. (The reference says: “This is the formal withdrawal of one or more papers by one or all of the authors. In most circumstances, retraction happens when new findings, or an inability by other groups to replicate results, spur the authors to withdraw a paper.”)

*We thank the authors for going through our Introduction and References meticulously. We have now changed the first sentence of our Introduction as per their suggestion: “Retraction refers to the formal withdrawal of a peer-reviewed publication, most often due to scientific misconduct or an error that invalidates the purported conclusions [1].”*

Page 4: “These results align well with broader studies,” The three citations refer to specific cases or a medical specialty – which do not count as “broader studies.” Broader studies to cite might be: https://asistdl.onlinelibrary.wiley.com/doi/full/10.1002/pra2.2016.14505301055 (disclosure: one of the reviewers is a co-author), or https://asistdl.onlinelibrary.wiley.com/doi/10.1002/pra2.35. There are others.

*Thank you very much for the citations, which we found extremely helpful. We have now updated the this sentence on Page 4 to read: “These results align well with literature on case studies (Bar-Ilan and Halevi 2017; Kochan and Budd 1992), specific disciplines (Rapani et al. 2020) and the broader literature (Budd et al. 2016), which identify that at least 80% of retracted articles receive positive post-retraction citations.”*

Page 5: “authors of guidelines and meta-analyses seem to very rarely update their work to remove retracted articles [10,11]” Citation 10 is a review (not research) article – it would be a secondary source at best, but it does not discuss guidelines or meta-analyses. In addition, while an author may request revisions, corrections or otherwise, a journal/publisher may refuse to allow them, so perhaps taking “authors” out of the equation and simply saying these are very rarely updated would be more accurate.

Page 5: “We hereby integrated the Retraction Watch Database..” Minor point, but “hereby” seems needlessly formal and can be deleted.

*Thank you very much for providing comments to improve the readability of our manuscript. We have now removed the “hereby” and the new sentence reads: “We hereby integrated the Retraction Watch Database …”.*

**Results**

Page 6: “Out of 4,217 research articles, 3,972 (94.2%) received a retraction notice (Table 1; S2 Table). This needs some context, and we would refer the authors to question 11 of the Retraction Watch Database User Guide: https://retractionwatch.com/retraction-watch-database-user-guide/ Corrections and Expressions of Concern (EoCs) have been entered as a point of interest related to authors with retractions. Unlike with retractions, there is no intention to be comprehensive on other types of citations.

*Indeed, we agree with the reviewers. We have now removed all mentions of such notifications and we have replaced the first sentence of our Results on Page 6 with: “As of August 21, 2020, the RetractionWatch database contained 22,200 publications with a unique Digital Object Identifier (DOI), PubMed ID (PMID) or title (when DOI or PMID were not available), published between 1923 and 2020.”*

Page 8: “It is unclear whether higher rates of retraction in certain journals/countries are due to less robust research practices versus more robust efforts to identify papers that should be retracted or confounded by other factors (e.g. type and impact of research done)” This comment seems more appropriate in a discussion section, and would be improved with some citations or evidence to support the speculation.

*This sentence was primarily meant to help readers appreciate that the cause behind the observed rate of retraction across countries is complex and that they should not misinterpret higher rates as evidence of less robust research. Given the confusion caused by this sentence, we have now removed it.*

Page 11: “The difference is rather balanced for 2,843 articles that are not popular - the extreme negative values around -400 came from a single retraction in Tumor Biol.” Was this the retraction notice that pulled more than 100 articles at one time? This would make the weighting of this notice very unequal to other “single article” notices.

*This is a valid concern. This is why (a) we have clearly indicated that retraction notices can refer to more than one original article (Page 9: “Out of 4,142 retraction notices with a unique DOI or PMID, 44 referred to the retraction of more than one original article - the largest retraction was published in Tumor Biology, retracting 103 unique articles due to fake peer review [15].”) and (b) why we have been using order statistics, like the median and the interquartile range, to describe our data to mitigate the impact of such outlier retractions. In addition, we have now also updated the identified sentence to add your remark: “The difference is rather balanced for 2,923 articles that are not popular - the extreme negative values at -395 came from a single retraction in Tumor Biol, which retracted 103 unique original articles.”*

Page 14: “2 articles only received non-negative tweets [16,17] .” Citation 16 and 17 belong to the articles presumably receiving “non-negative tweets”. Perhaps the authors could change it to “2 articles only (Wansink et al. and Larkin et al.) only received non-negative tweets [16,17]” to make it clear that the citations do not belong to a substantiation of the tweet numbers. The authors identify the two in the subsequent paragraph – but perhaps best to clarify it in the first sentence.

*We thank the reviewers for helping us improve the readability and clarity of our manuscript. We have now updated the sentence to the following: “2 articles exclusively received non-negative tweets [16,17].”*

Page 14: “Green signifies a non-negative tweet and red signifies a negative tweet.” Using colors as the sole indicator is problematic in readability for colorblind readers, which one of the reviewers is. Could the authors combine the colors with some other type of indicator – such as bolded letter vs non-bolded, stars and squares for data points instead of simply colors?

*Thank you very much for your input on how to make our manuscript more accessible. We have now updated the Figure and the sentence accordingly: “Green (bold) signifies a non-negative tweet and red in italics signifies a negative tweet.*

**Discussion**

Page 18: “At least half of retracted research articles received some amount of Altmetric attention.” Would suggest adding “studied” after “articles” to clarify the sample analyzed.

*We have now modified the sentence, as suggested, into: “At least half of retracted research articles studied received some amount of Altmetric attention.”*

Page 19: “Indeed, in one of our sensitivity analyses we surprisingly noticed that in two of the articles examined, retraction led to the promotion of the initial misinformation (by being followed by further tweets spreading the initial results), rather than correcting the record.” We would suggest adding an example to demonstrate this.

*The tweets associated with both of these articles were described within the text and in Figure 6. However, the PLOS One editor alerted us to the fact that an approval is needed to include such tweets verbatim, for which reason we have now removed these tweets from our manuscript, as all relevant information is contained within the text. Despite the additional constraint, we have now reworded this sentence in response to your feedback: “Indeed, as we describe above, in one of our sensitivity analyses we surprisingly identified that in two of the articles for which post-retraction tweets were examined, retraction led to the promotion of the initial misinformation (by being followed by further tweets spreading the initial results), rather than correcting the record.”*

Page 19: “PubMed has been flagging retractions for years and it recently introduced a larger banner to help make retractions more apparent [28].” The authors may want to note that the banner effect is only available when the journal/publisher sends the retraction information in an appropriate format so the articles are linked. Retractions are still showing up without the original article being flagged, based on the metadata and information provided by the journal/publisher.

*Thank you very much for contributing this detail to us. We have now modified the document to: “PubMed has been flagging retractions for years and it recently introduced a larger banner to help make retractions more apparent [28], even though this banner only appears when the journal/publisher provides appropriate meta-data about the retraction to PubMed.”*

Page 20: “Finally, a number of inconsistencies were identified while working with the data extracted from the Retraction Watch Database (see Methods). Even though we tried to correct as many of these as we could, it is possible that more of these exist.” It is deeply problematic to include this comment without noting that the authors chose to scrape a database clearly marked “beta” at the time, and did not contact the (well-known) creators of the database to either request a full download -- which is made freely available to scholars subject to a simple data use agreement -- or query the inconsistencies. The “beta” designation was not removed until October 2018, when the database was officially launched: https://www.sciencemag.org/news/2018/10/what-massive-database-retracted-papers-reveals-about-science-publishing-s-death-penalty As noted in our general comments, we would strongly recommend that the authors now request a download and repeat their analyses. If they choose not to do so, the manuscript requires prominent notices that the authors chose to use data marked “beta” that was known to be incomplete at the time.

*As stated above and in personal communications, we deeply apologize for, unbeknownst to us despite notifications on the website, using a beta database and not requesting the data from the creators themselves. We have now signed a data agreement with the creators, updated our manuscript and analyses to reflect the new data and removed the aforementioned sentence from our Limitations section.*

**Methods**

Page 22: “It was made available to the public in October, 2018 [2] … We extracted all records available on this database as of May 29, 2018 that were originally published between 2010-2015.” This sentence is somewhat confusing. It suggests, as we have noted elsewhere, that the authors extracted all the data prior to the “beta” designation being removed. If so, how did they extract it? Or did they extract it after the database went public, in which case it is unclear how they would know what was available on May 29, 2018? Did they have permission to acquire the information, as Retraction Watch has data usage requirements?

If the data were extracted prior to the public data, then they were not complete, in which case the data and thus the analyses/results are based on incomplete and potentially biased data without being marked that way. While no resource will ever be 100% comprehensive for a variety of reasons that make retraction notices very difficult to find (e.g., notice in print form only and yet to be discovered, foreign language issues, journals removing articles from Table of Contents without notices, etc.), the authors have exacerbated these issues without noting that limitation in their manuscript, instead referring without comment to inconsistencies.

Also, cross-checking the database for retractions made up to May 29, 2018, using Article type: Research Article or Clinical Study or Letter, and restricting the dates of publication from 01/01/2010 to 12/31/2015, only 4108 entries are returned. Again, the data until this point were incomplete, and the authors extracted data before a “study” was completed and are using the data prematurely.

*As indicated above, we deeply apologize for, unbeknownst to us, using a beta database and not requesting the data from the creators themselves. We are now using data acquired using a data use agreement with the creators, re-analyzed the data and updated our manuscript accordingly.*

*We have now changed the first sentence of Data acquisition on page 22 to: “This is a retrospective cohort study of the retracted literature found in the Retraction Watch Database shared with us under a data use agreement on August 21, 2020.” We have also added the following in our Data Availability statement: “The Retraction Watch Database is available from Retraction Watch and requests for this data should be sent to: team@retractionwatch.com.”*

Page 23: “A publication was considered a research article when labelled by Retraction Watch Database as any of: Research Article, Clinical Study, Clinical Study/Research Article, Letter or Letter/Research Article.” The number of retractions was compared with the number of publications per year, using the NSF counts (ref 36), but the NSF excludes letters in their counts: “The articles exclude editorials, errata, letters, and other material that do not present or discuss scientific data, theories, methods, apparatuses, or experiments. The articles also exclude working papers, which are not generally peer reviewed.” Did the authors confirm that the retractions associated with “Letters” would have been included in the NSF counts?

*This is very perceptive and we thank the reviewers for this comment. We included letters in our definition of research articles because such articles often (but certainly not always) include empirical research. In our study of 4,603 unique research articles, 21 were letters that would not have been included had we not included “Letters” within our definition of research articles (0.5%). As such, we do not believe that inclusion of letters invalidates standardization by NSF counts of peer-reviewed documents. Nevertheless, this is an important clarification and we have included the following sentence in the legend of Figure 1, which uses NSF counts: “Note that NSF counts of total peer-reviewed documents do not include letters, which represent 21/4,603 (0.5%) of our research articles as per our eligibility criteria.”*

Page 23: “In 37/3,972 research articles, the reported date for the original article was paradoxically later than the reported date for its retraction - we excluded these articles when reporting on the duration between publication of the original and its retraction.” Why did the authors not just attempt to locate the correct dates and then include the articles? Did they consider contacting the creators of the database to alert them to the pre-launch errors?

*We thank the authors for alerting us to the availability of the correct Retraction Watch Database and for sharing this with us. Such dates were no longer an issue and all of the paragraph within which this sentence was found has been deleted.*

Page 23: “Altmetric indicates that articles with an AAS > 20 are thought to roughly represent the top 5% of the literature” Citation?

*This number was given to us in a personal communication. We now refer readers to a public indication that an article with AAS > 20 is performing much better than its contemporaries at the following link:* [*https://tinyurl.com/yxnqq2qt*](https://tinyurl.com/yxnqq2qt)*. We have accordingly modified the quotes sentence into: “Altmetric indicates that articles with an AAS > 20 are thought to be doing “far better than their contemporaries” [citation] - we call articles with an AAS > 20 in our sample “popular”.*

Page 26: “we automatically identified a maximum of 5 random unretracted full articles from the same issue and journal. We indicate a “maximum of 5” as we could not always find 5 unretracted articles in the same issue.” The concept of randomness is fine - except when the selections for control are so disparate. Perhaps the authors could separate journal issues into groups of comparable sizes, then choose random samples from each.

*We appreciate the concern of the reviewers. However, from a statistical theoretic point of view, such differences in number of controls do not invalidate the study design or randomization. In fact, a design where every case is matched to a single control would exacerbate any uncertainty in the underlying effect, rather than mitigate it. More or less controls do not introduce bias in calculating the effect size, which in this case refers to the difference in AAS. Less controls do increase the uncertainty in our estimate of effect, but this uncertainty was calculated and incorporated within our hypothesis testing procedure. With as many as 450 distinct groups, we are confident in the results presented.*

*To empirically illustrate the above point, we stratified our groups by inclusion within journals that typically publish many articles of different disciplines vs. not. By inspecting all journals applicable to 2 or more groups, we identified the following journals: PLOS ONE (9 groups), Nat Commun (3 groups), Nature (3 groups). We then rerun our analysis stratified by inclusion within such journals of “broad” interest. As you can clearly see in the table below, the direction of effect within both strata is the same one identified in the unstratified analysis, namely that retracted articles tend to have a larger AAS than matched unretracted articles.*

Page 25: “we examined all tweets gathered by Altmetric for all popular articles retracted within a year since we downloaded our Altmetric data.” As Altmetric uses multiple sources (blogs, tweets, various main and social media sources), using tweets as an indication of the positive or negative measure of attention seems a bit narrow. Users of Twitter is a very biased group in itself (e.g. https://www.pewresearch.org/internet/2019/04/24/sizing-up-twitter-users/), may actually not be the widest used social media commenting platform, (https://blog.hootsuite.com/twitter-demographics/), and may not be appropriately comparable to the measure of attention an article is receiving across main media outlets or filed-related blogs. Did the authors cross-reference their Twitter findings against other platforms and genres?

*This is indeed a legitimate concern and an in-depth analysis of attention received by retracted literature across different media would be of great interest as a follow up study. However, from our own experience, for most articles (not all), tweets are the primary source of attention. This is also the case for both articles we examined in depth, both of which received a substantial number of tweets, but very little attention elsewhere. Nevertheless, we agree that readers should appreciate that AAS is a multi-factorial metric and that different media may suffer from their own biases. For this reason, we have modified the quoted sentence into: “we examined the tweets associated with all 17/317 recently retracted articles that were popular even before retraction (317 instead of 279 because all original articles with a DOI were considered; also, note that tweets only constitute part of the AAS, which is a multi-factorial metric).*

Page 27: “for 2,944/10,370 (14.5%) records the database did not offer any of the two and did not offer any other unique identifier” The database does not issue these numbers -- PubMed and CrossRef do, when publishers apply for them -- and would only have them if they were available. Perhaps language change to “No PMID or DOI were available for 2,944/10,370 (14.5%) records in the database, which relies on these two external and common identifiers.”

*We have now implemented this feedback and updated this sentence into: “Even though it is possible to identify notices referring to the same original article by using the PMID or DOI, for 140/4,6011 (3%) records the database did not offer any of the two external and common identifiers. We have also removed the sentence “and did not offer any other unique identifier” as this is now not true.*

# Reviewer 2

This was an interesting work examining the Altmetric Attention Score for retracted papers both before and after retraction. The authors chose 2010-2015 works from RetractionWatch and grabbed data from Crossref, PubMed and Altmetric.com to supplement information. My small concern that the authors should address is that Altmetric.com started tracking attention Oct, 2011. Thus, articles published before Oct 2011 could have misleading AAS and this needs to be addressed in the paper.

The statistics, supplemental information, and overall stats seem appropriate, but I am not an expert on statistics and would defer to other peer reviewers with more knowledge in this area to determine if other measures could/should have been used.

Overall, I found the work to be well written and logical. The information visualizations seem appropriate and paint the picture the authors are telling.

*Thank you very much for your kind words.*

I would consider focusing a bit more on how positive/negative tweets were analyzed; was there a sentiment analysis performed and how was it performed? Was it just based on authors' impressions? More clarity here, please. This is of interest because it suggests that Twitter users were aware of the retraction and posted messages to help others become aware. Were these tweets warnings to others? Did they call out misinformation spread? There are plenty of articles performing sentiment analysis on tweets and the authors should perform a quick review and specify what is applicable to tweets and what is not (e.g., [Social Web Sentiment Analysis by Mike Thelwall](http://ucrel.lancs.ac.uk/crs/attachments/UCRELCRS-2013-05-16-Thelwall-Slides.pdf)).

*Thank you very much for your insightful feedback. This analysis was based on our own impressions. We have now modified our Materials and Methods section on Page 27 to reflect that: “We then counted how many tweets were tweeted before and after retraction, how many of the pre- and post-retraction tweets we perceived based on our own impression as negative (e.g. “this article has now been retracted”) or non-negative (e.g. “this article presents impressive results”) and copied the first negative and the first non-negative pre- and post-retraction tweet (always the first to avoid bias)”.*

*We do not believe that our findings suggest that the Twitter users were necessarily aware of the retraction. Indeed, on two occasions there was no single post-retraction tweet that referenced the retraction. We have now made this point clearer by noting the following in our Discussion (Pages 20-21): “Indeed, as we describe above, in one of our sensitivity analyses we surprisingly identified that in two of the articles for which post-retraction tweets were examined, retraction was directly or indirectly associated with the promotion of the initial misinformation (by being followed by further tweets spreading the initial results), rather than correcting the record.*

*Indeed, misinformation spread is of major interest to us and studying attention received across different media in more detail is a compelling follow up study. In the current manuscript, we systematically examined the first tweet before and after retraction - even though we would have loved to report all tweets we examined verbatim within the manuscript, the editor alerted us to the fact that this is not permissible - these tweets can still be found in our OSF account, where all of the data we extracted has been stored.*
