# Supplementary material for: Media and social media attention to retracted articles according to Altmetric
Source: PLoS One. 2021 May 12;16(5):e0248625. doi: 10.1371/journal.pone.0248625 (PMC8115781; doi:10.1371/journal.pone.0248625)
Supplement: Attachment — Submitted filename: response-to-reviewers.docx [file pone.0248625.s010.docx]

# Reviewer 1

We appreciate the authors' responsiveness to previous comments. We only have a few minor comments to add:

1. In Methods, the authors note: “we extracted the total number of peer-reviewed documents”, and in the following paragraph “peer-reviewed” was added to the statement: “All research articles on the Retraction Watch Database that were originally published between 2010-2015...” The authors further added: “Preprints and dissertations were not included because they are not peer-reviewed publications.” Did the authors confirm that all the articles in their sample were indeed from peer-reviewed journals as there is no filtering requirement for peer-reviewed journals for inclusion in the database?

*Thank you for this comment. Yes, we confirmed this was the case by selecting for the research article types we noted in the Methods. However, upon your comment, we noted that some articles were labelled as both “Research Article” and “Preprint”. We have gone ahead to relabel those articles as not peer-reviewed and have updated our code on GitHub accordingly - however, despite the change, none of those articles had been eligible for our work, so no changes in the quoted numbers were necessary. We have reworded our methods to clearly indicate that “Preprints and dissertations were excluded because they are not peer-reviewed publications.”*

2. We previously commented about a paragraph from Page 5 in the prior manuscript: “Page 5: “authors of guidelines and meta-analyses seem to very rarely update their work to remove retracted articles [10,11]” Citation 10 is a review (not research) article – it would be a secondary source at best, but it does not discuss guidelines or meta-analyses. In addition, while an author may request revisions, corrections or otherwise, a journal/publisher may refuse to allow them, so perhaps taking “authors” out of the equation and simply saying these are very rarely updated would be more accurate.” There are no author comments regarding this, although the citation now is changed to “11,12.” Again, citation (now) 11 is a review article and does not discuss guidelines and meta-analyses in particular. And we reiterate that authors may have little control over the updating of their work. So again – perhaps modifying the reference choice and changing the language to remove the perceived burden of updating being solely that of the authors would be more appropriate.

This is a very perceptive comment and we agree with the reviewer. We have now reworded the appropriate phrase into “guidelines and meta-analyses seem to be very rarely updated to remove retracted articles”. We have also now removed citation 11 and only retained citation 12 (which is now, citation 11).

3. The data availability section for the manuscript acceptance says ”yes- all data are fully available without restriction.” However, elsewhere the authors note (correctly) that data from the Retraction Watch Database are available from the Center For Scientific Integrity. We would suggest ensuring that these responses are consistent.

*We apologize for the former, which was our mistake in the process of submitting our manuscript. We will make sure that we select the choice that reflects the data availability statement of our manuscript when we resubmit.*
